# Evaluation of Peroxidase in Herbal Medicines Based on an Electrochemical Sensor

**DOI:** 10.3389/fchem.2021.709487

**Published:** 2021-06-23

**Authors:** Yinzi Yue, Lianlin Su, Min Hao, Wenting Li, Li Zeng, Shuai Yan

**Affiliations:** ^1^ First Clinical Medical School, Nanjing University of Chinese Medicine, Nanjing, China; ^2^ School of Pharmacy, Nanjing University of Chinese Medicine, Nanjing, China; ^3^ School of Pharmacy, Zhejiang Chinese Medicine University, Hangzhou, China; ^4^ Department of Anorectal, Suzhou TCM Hospital Affiliated to Nanjing University of Chinese Medicine, Suzhou, China

**Keywords:** peroxidase activity, herbal medicines, electrochemical evaluation, rapid detection, hydrogen peroxide

## Abstract

Peroxidases are species-specific. Differences in peroxidase can objectively reflect the genetics among species. The use of peroxidase to assist in species identification is relatively simple and effective. In this work, we proposed a graphene-modified electrode. This electrode can amplify the signal of electrocatalytic reduction of hydrogen peroxide. Since peroxidase can catalyze the reduction of hydrogen peroxide, this signal can be used as an indicator to demonstrate the content of peroxidase in different plant tissues. Twelve herbal medicines were selected for our study. The results show that this electrochemical-based detection technique was comparable to colorimetric method in terms of accuracy.

## Introduction

The phenolics and enzymes in plant tissues are distributed in different locations in the cell. Enzymes cannot come into contact with phenolic substrates, so enzymatic browning does not occur (Paravisini and Peterson, 2019; Kizilgeci et al., 2020; Moon et al., 2020; Özkan, 2020). However, if damage or dehydration leads to the destruction of plant cell structure and changes the regionalized distribution of phenolics and enzymes, the contact between phenolic substances, enzymes and oxygen will cause enzymatic browning (Barpete et al., 2020). The presence of phenolic substrates, enzymes and oxygen is necessary for enzymatic browning to occur. Enzymes that cause enzymatic browning of plant include polyphenol oxidase, peroxidase, catalase, superoxide dismutase (Demir and Işık, 2020), phenylalanine aminotransferase, etc. Among them, peroxidase is the main oxidase that causes enzymatic browning in most plants (Ozbek et al., 2021; Tenish et al., 2021).

Peroxidase is an oxidase that uses hydrogen peroxide as an electron acceptor to catalyze substrates and is widely found in plants, animals, and microorganisms (Chen et al., 2020; Karimi-Maleh et al., 2021a; Lazzarotto et al., 2021). Most peroxidases contain heme cofactors and are mostly heme-binding proteins containing iron ions. There are also some peroxidases in which the iron of heme is replaced by copper, manganese, vanadium or selenium (Dong et al., 2020; Karimi-Maleh et al., 2021c). Therefore, according to the different cofactors, ferric heme peroxidases can be divided into two categories. The third type of structure is the chloride peroxidase and the cytochrome c peroxidase containing two hemoglobins (Fu et al., 2019; Li et al., 2020; Zhou et al., 2020). In addition, depending on the isoelectric point, it can be divided into acid peroxidase, neutral peroxidase and basic peroxidase. According to the binding state, it can be divided into soluble peroxidase, ion-bound peroxidase and covalent-bound peroxidase (Xu et al., 2020; Karimi-Maleh et al., 2021b).

The activity and number of peroxidases vary greatly in different tissues and organs, different growth, development periods, different physiological states and different varieties of plants. Peroxidases can largely reflect the characteristics of plant growth and development, biometabolic status, ability to adapt to the external environment and genetic differences among varieties. The electrophoretic profiles of peroxidases are relatively stable under certain conditions and are as species-specific as morphological trait indicators (Yang et al., 2020; Ying et al., 2020; Zheng et al., 2020). It has been widely used as a genetic marker in plant variety identification, genetic diversity analysis, plant disease resistance analysis, plant growth and development analysis (Karimi-Maleh et al., 2020).

Non-denaturing discontinuous polyacrylamide gel electrophoresis (native PAGE) does not easily denature proteins. It essentially does not disrupt the natural conformation of proteins and the subunit interactions, so it maintains protein biological activity (Rajhans et al., 2020; Almaz et al., 2021). This method is most widely used in the detection of plant peroxidases because it does not denature proteins. Sodium dodecyl-sulfate-gelatin-poly-acrylamide gel electrophoresis (G-PAGE) is an electrophoretic technique established in the early 1980s, which is a kind of electrophoresis that maintains the enzyme biological activity after electrophoresis (Liu et al., 2020). In phycological studies, peroxidases are species-specific. Differences in peroxidases can objectively reflect the genetics among species. The use of peroxidase to assist in species identification is a relatively simple and effective. Studies have shown that peroxidases are involved in the physiological responses of plants to disease, insect, salt, and drought resistance as well as resistance to biotic stresses (Jamali et al., 2014; Baghizadeh et al., 2015; Karaman, 2021; Karaman et al., 2021).

Recently, an electrochemical-based detection technique has been developed for the study of peroxidase activity in plants (Fu et al., 2021). Since peroxidase can catalyze hydrogen peroxide accordingly, differences in catalytic activity can be used to reflect differences in peroxidase content in plant tissues. This method can potentially be used for the determination of plant sex. Electrochemical detection is a low-cost and university-based analytical technique that is particularly suitable for rapid detection. Therefore, this technology has a bright future in plant detection. There is a very large market for herbal medicine in Asia. Authentication of herbal medicines has been a problem in this market. Based on the above information, the identification of herbal medicines by using the difference of peroxidase is a direction worth exploring.

In this work, twelve herbs were selected for electrochemical testing. The peroxidases in the herbs were first extracted. Graphene-modified electrodes were subsequently used to detect these extracts in the presence of hydrogen peroxide. The results revealed a large variation of peroxidase in different herbs. This technique can potentially be used for the identification of herbal species as well as the control of herbal quality.

## Experiments

### Materials

Ratan of *Erycibe obtusifolia*, leaf of *Panax ginseng* C. A. Mey, leaf of *Murraya exotica* L., rhizome of *Zingiber officinale* Rosc, seed of *Cassia obtusifolia* L., rhizome of *Pinellia ternate* (Thunb.) Breit, rhizome of *Imperata cylindrica* Beauv. var.major (Nees) C.E.Hubb, pericarp of *Zanthoxylum bungeanum* Maxim, seed of *Trichosanthes rosthornii* Harms, pericarp of *Benincasa hispida* (Thunb.) Cogn, rhizome of *Semiaquilegia adozoides* (DC.) Makino and seed of *Strychnos nuxvomica* L. were purchased from local pharmacy and identified by Nanjing University of Chinese Medicine. Disodium hydrogen phosphate, sodium dihydrogen phosphate, potassium dihydrogen phosphate, guaiacol, hydrogen peroxide, graphene ink, sulfuric acid, phosphoric acid, anhydrous ethanol, Thomas Brilliant Blue G-250, bovine serum albumin were all analytically grade.

### Peroxidase Extraction

Weigh a certain amount of herbs and add PBS at 4°C. The pulping time was 6 0°s, and then extracted for a certain time at 4°C. The filtered filtrate was frozen and centrifuged at 9,000°r/min for 15°min, and the supernatant was collected as the crude enzyme solution.

### Enzyme Activity Measurement Based on Colorimetric Assay

Guaiacol was used as the reaction substrate in the colorimetric assay. The reaction system consisted of 2.95 ml of 18 mM guaiacol and 1 mM H_2_O_2_ (pH 5 PBS). Add 0.05 ml of the enzyme solution, cover the cuvette with a lid and mix rapidly, measure the absorbance value at 470 nm at 30°C, count one time every 10°s, and use 0.01 change in absorbance value per minute as 1 unit of enzyme activity.

### Determination of Protein Content

The protein content was determined by the colorimetric method of Bradford’s Komas Brilliant Blue G-250. Bovine serum protein was used to make the standard curve. The absorbance value at 595 nm was used as the vertical coordinate for the standard curve. The standard curve was plotted with the standard protein content as the horizontal coordinate, and the protein mass in the sample was calculated by the curve equation. Specific activity is the activity per unit mass of enzyme, expressed as U/mg, specific activity = activity (U)/mass of protein (mg).

### Enzyme Activity Measurement Based on Electrochemical Method

All electrochemical measurements were performed using a CHI 820D electrochemical workstation with a three-electrode system. Specifically, a reference electrode (Ag/AgCl), a counter electrode (Pt foil) and a working electrode (glassy carbon electrode, GCE). Graphene ink (0.5 mg/ml) was firstly drop coated on the GCE surface and dried naturally. Then, a linear sweep voltammetry was used for detecting the electrocatalytic of peroxidase toward H_2_O_2_.

## Results And Discussion

**FIGURE 1:**
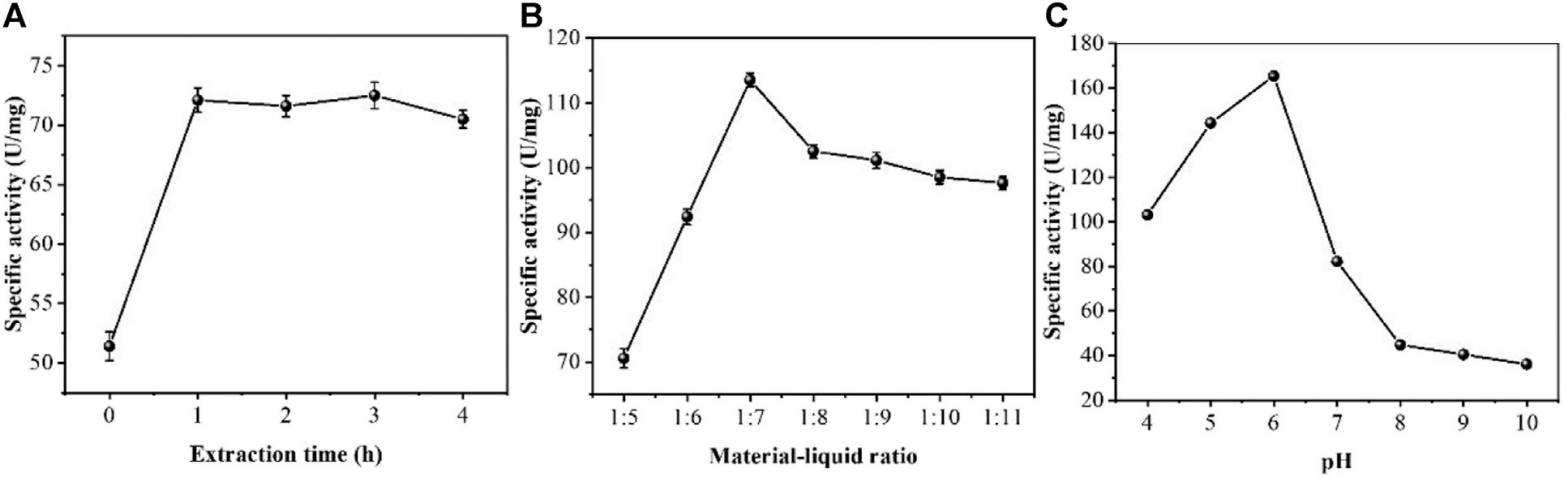
Effect of **(A)** extraction time **(B)** material-liquid ratio and **(C)** pH on enzyme specific activity of *Erycibe obtusifolia.*

Firstly, we optimized the extraction process of peroxidase. The extraction conditions were optimized using an orthogonal test. The results of the single-factor test were analyzed in an orthogonal test to derive the key factors influencing the peroxidase extraction of *Erycibe obtusifolia*. The effects of different extraction times of 0, 1, 2, 3 and 4 h on the specific activity of *Erycibe obtusifolia* peroxidase were investigated by fixing the material-liquid ratio 1:5 at pH 4. It can be seen from Figure 1A that the enzyme specific activity increased with the increase of extraction time and stabilized when the extraction time exceeded 1 h. The reason may be that the enzyme in *Erycibe obtusifolia* tissue was not fully solubilized when the extraction time was less than 1 h. With the increase of time, the enzyme leaching gradually reached the equilibrium, and the enzyme specific activity tended to stabilize. 3 h later, the enzyme specific activity slightly decreased, which might be due to the inactivation of the leached enzyme in the solution environment. Figure 1B examines the effect of different stock-to-solution ratios on the specific activity of honeysuckle peroxidase. The peroxidase specific activity increased with the increase of the material-liquid ratio between 1:7 and 1:5. When the material-liquid ratio was less than 1:7, the peroxidase specific activity showed a decreasing trend with decreasing material-liquid ratio. This may be due to the increase of solids in the extraction system and the relative lack of extraction solution due to the large material-liquid ratio. When the material-liquid ratio was smaller, the enzyme leached from plant tissues was diluted and showed a decrease in enzyme activity. Figure 1C examines the effect of different extract pH values on the specific activity of *Erycibe obtusifolia* peroxidase. From Figure 1C, it can be seen that the enzyme specific activity showed a trend of increasing and then decreasing between pH 4–9. The buffer pH of six had a better extraction effect. The reason may be that the alkaline and more acidic environment had an effect on the conformation of *Erycibe obtusifolia* peroxidase, which led to a change in the molecular structure of the enzyme causing a partial loss of enzyme activity. On the other hand, it may be that more heteroproteins were leached at pH greater than 6, thus affecting the peroxidase extraction effect.

**FIGURE 2:**
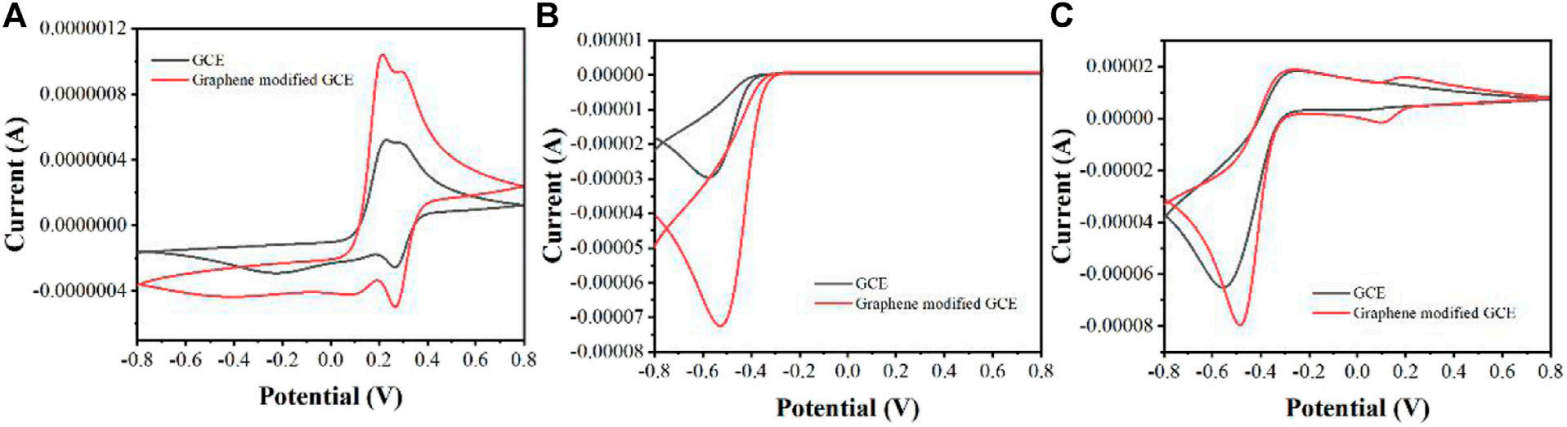
**(A)** CVs of GCE and graphene modified GCE on *Erycibe obtusifolia* peroxidase extracts in the absence of H_2_O_2_ **(B)** CVs of GCE and graphene modified GCE toward 1 mM H_2_O_2_ **(C)** CVs of GCE and graphene modified GCE on *Erycibe obtusifolia* peroxidase extracts in the presence of 1 mM H_2_O_2_.

Figure 2 shows the CV curves of GCE and graphene modified GCE on *Erycibe obtusifolia* peroxidase extracts. In the absence of H_2_O_2_ (Figure 2A), both electrodes have some redox peaks during the anodic scan and cathodic scan. These peaks are due to the oxidation and reduction of some substances possessing electrochemical activity in *Erycibe obtusifolia* extracts, such as flavonoids (Cai et al., 2020), pigments (Pearce et al., 2020), carotenoids (Čižmek and Komorsky-Lovri), etc.

Figure 2B shows the CV curves of the two electrodes in PBS for 1 mM H_2_O_2_. It can be seen that both electrodes have a reduction of H_2_O_2_ in the cathodic scan. In contrast, the graphene-modified GCE possesses a very remarkable current signal. Therefore, graphene can significantly increase the current signal due to its excellent electrical properties (Vasseghian et al., 2021).

The same can be observed in the assay of *Erycibe obtusifolia* extracts (Figure 2C). It can be seen from the figure that the graphene-modified electrode possesses a very significant current signal. This amplification strategy of the current signal can greatly increase the feasibility of detecting peroxidase by electrochemistry.

**FIGURE 3:**
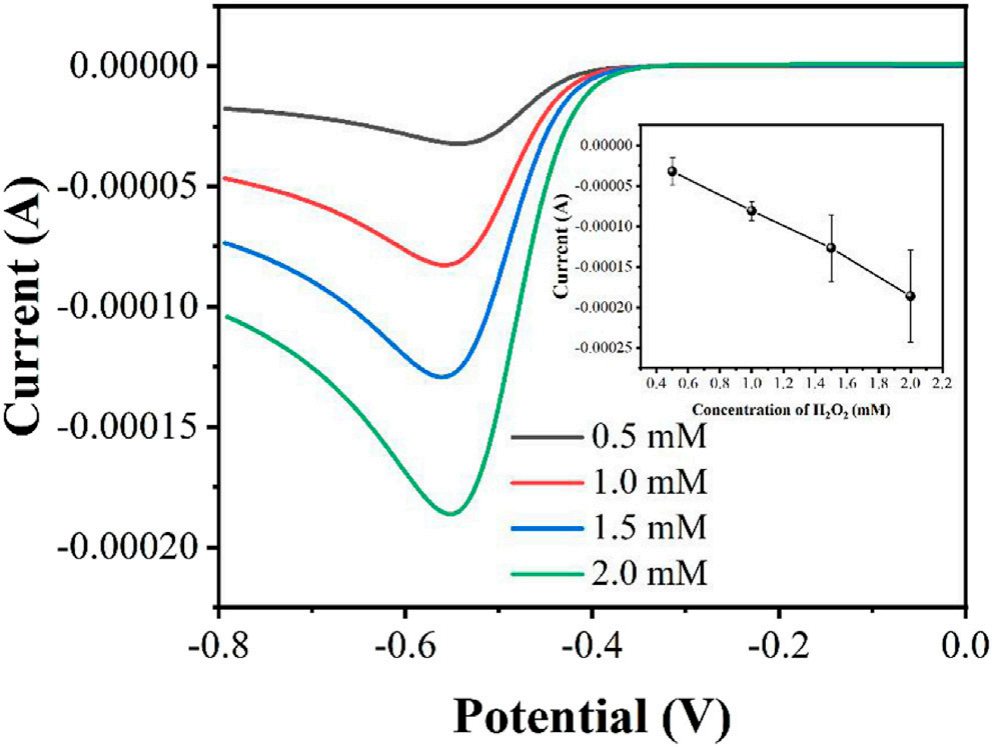
Effect of the H_2_O_2_ concentration on the determination performance (*n* = 3).

The concentration of H_2_O_2_ affects the performance of electrocatalytic response. In general, the higher the concentration of H_2_O_2_ will lead a higher reduction current. However, too much H_2_O_2_ can rapidly deplete the peroxidase enzyme, thus leading to a large variance. Figure 3 shows the LSV curves in the presence of 0.5, 1, 1.5 and 2 mM H_2_O_2_. As expected, the reduction current increases with increasing concentration of H_2_O_2_. However, the reproducibility of the currents becomes worse as the concentration increases. Therefore, we finally chose 1 mM H_2_O_2_ as the experimental concentration.

**FIGURE 4:**
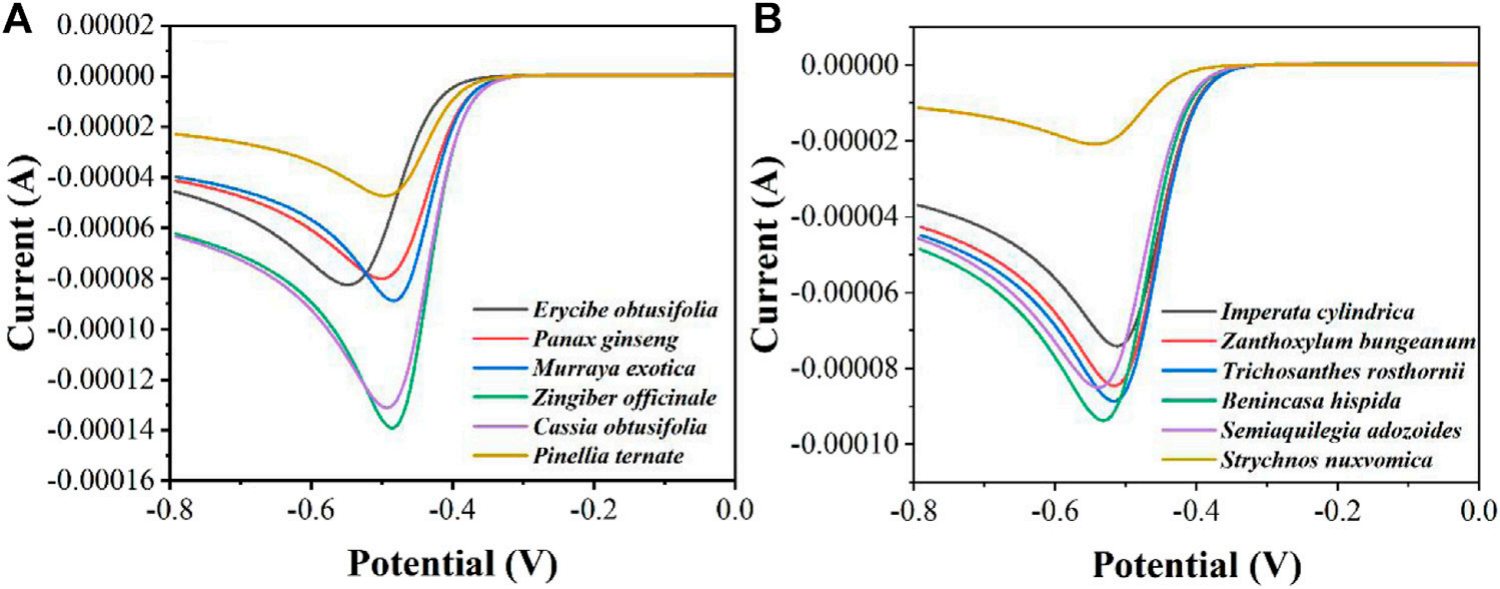
LSV curves of **(A)** *Erycibe obtusifolia, Panax ginseng, Murraya exotica, Zingiber officinale, Cassia obtusifolia, Pinellia ternate* and **(B)** *Imperata cylindrica, Zanthoxylum bungeanum, Trichosanthes rosthornii, Benincasa hispida, Semiaquilegia adozoides*, *Strychnos nuxvomica* in the presence of 1 mM H_2_O_2_ recorded using a graphene modified GCE.

In order to distinguish more quickly the differences in peroxidase in different herbal extracts, we measured twelve herbs by LSV (Figure 4). It can be seen that *Zingiber officinale* has the highest current and *Strychnos nuxvomica* has the lowest current. According to the data in Figure 3, the order of peroxidase activity of the twelve herbs was *Zingiber officinale* > *Cassia obtusifolia > Benincasa hispida > Murraya exotica > Trichosanthes rosthornii > Semiaquilegia adozoides > Zanthoxylum bungeanum > Erycibe obtusifolia > Panax ginseng > Imperata cylindrica > Pinellia ternate > Strychnos nuxvomica*. Table 1 shows the results of 12 herbs tested by colorimetric assay. Except for *Zanthoxylum bungeanum* and *Semiaquilegia adozoides*, the order of the results is consistent with that of the electrochemical assay. Such results represent that the electrochemical detection techniques we use can be used for the identification and quality control of herbs.

**TABLE 1:** Enzyme activity measurement of *Erycibe obtusifolia*, *Panax ginseng*, *Murraya exotica*, *Zingiber officinale*, *Cassia obtusifolia*, *Pinellia ternate*, *Imperata cylindrica*, *Zanthoxylum bungeanum*, *Trichosanthes rosthornii*, *Benincasa hispida*, *Semiaquilegia adozoides* and *Strychnos nuxvomica* based on colorimetric assay.

Herb	Specific activity (U/mg)	Herb	Specific activity (U/mg)
*Erycibe obtusifolia*	162	*Panax ginseng*	154
*Murraya exotica*	174	*Zingiber officinale*	197
*Cassia obtusifolia*	181	*Pinellia ternate*	72
*Imperata cylindrica*	149	*Zanthoxylum bungeanum*	169
*Trichosanthes rosthornii*	152	*Benincasa hispida*	178
*Semiaquilegia adozoides*	167	*Strychnos nuxvomica*	31

## Conclusion

Peroxidase can be used as an indicator for the identification of herbs and quality control. An electrochemical-based assay was proposed for the rapid detection of peroxidase in herbal medicines. Graphene was used to modify the electrode to achieve increased signal sensitivity. Peroxidase in herbs can catalyze the electrochemical reduction of H_2_O_2_, so the electrochemical reduction signal of H_2_O_2_ can be used as an indicator for the content of peroxidase in the samples. Based on a investigation of 12 herbs, the accuracy of this detection technique is comparable to that of colorimetric method.

## Data Availability

The original contributions presented in the study are included in the article/Supplementary Material, further inquiries can be directed to the corresponding authors.
